# Supplementary material for: The Systems Biology Research Tool: evolvable open-source software
Source: BMC Syst Biol. 2008 Jun 29;2:55. doi: 10.1186/1752-0509-2-55 (PMC2446383; doi:10.1186/1752-0509-2-55)
Supplement: Additional file 1 — SBRT Archive. An archive of the current version of the Systems Biology Research Tool. [file 1752-0509-2-55-S1.zip › sbrt-1.4.0/doc/developers_guide/api/sbrt/kernel/util/UnmodifiableSet.html]

UnmodifiableSet


|  |  |  |
| --- | --- | --- |
| **Overview** \| **Package** \| **Class** \| **Use** \| **Tree** \| **Deprecated** \| **Index** \| **Help** | |  |
| **PREV CLASS**   **NEXT CLASS** | **FRAMES**    **NO FRAMES**     **All Classes** |  |
| SUMMARY: NESTED \| FIELD \| CONSTR \| METHOD | DETAIL: FIELD \| CONSTR \| METHOD |  |


---


## sbrt.kernel.util Class UnmodifiableSet<E>

```
java.lang.Object
  sbrt.kernel.util.UnmodifiableSet<E>
```

**Type Parameters:**: `E` - the element type.

**All Implemented Interfaces:**: java.lang.Iterable<E>, java.util.Collection<E>, java.util.Set<E>

---

``` public class UnmodifiableSet<E> extends java.lang.Object implements java.util.Set<E> ```

This class is used to create an unmodifiable view of sets. It
is intended to mimic the behavior of
`java.util.Collections.unmodifiableSet(Set)`,
but in a more explicit way. If a method returns an
`UnmodifiableSet`, it's clear that the set is
unmodifiable and that `UnsupportedOperationException` will be thrown
if modification is attempted.

**Author:**
:   This class was written and documented by Jeremiah Wright while
    in the Wagner lab.

---

**Constructor Summary**

`UnmodifiableSet(java.util.Set<E> set)`
  
          Constructs a new unmodifiable view of the provided set.


| **Method Summary** | |
| --- | --- |
| `boolean` | `add(E o)`             See Set.add(Object). |
| `boolean` | `addAll(java.util.Collection<? extends E> c)`             See Set.addAll(Collection). |
| `void` | `clear()`             See Set.clear(). |
| `boolean` | `contains(java.lang.Object o)`             See Set.contains(Object). |
| `boolean` | `containsAll(java.util.Collection<?> c)`             See Set.containsAll(Collection). |
| `boolean` | `equals(java.lang.Object obj)`             See Set.equals(Object). |
| `int` | `hashCode()`             See Set.hashCode(). |
| `boolean` | `isEmpty()`             See Set.isEmpty(). |
| `java.util.Iterator<E>` | `iterator()`             See Set.iterator(). |
| `boolean` | `remove(java.lang.Object o)`             See Set.remove(Object). |
| `boolean` | `removeAll(java.util.Collection<?> c)`             See Set.removeAll(Collection). |
| `boolean` | `retainAll(java.util.Collection<?> c)`             See Set.retainAll(Collection). |
| `int` | `size()`             See Set.size(). |
| `java.lang.Object[]` | `toArray()`             See Set.toArray(). |
| `<T> T[]` | `toArray(T[] a)`             See Set.toArray(Object[]). |
| `java.lang.String` | `toString()`             Returns `toString()` on the underlying set. |

**Methods inherited from class java.lang.Object**

`clone, finalize, getClass, notify, notifyAll, wait, wait, wait`

**Constructor Detail**

### UnmodifiableSet

```
public UnmodifiableSet(java.util.Set<E> set)
```

:   Constructs a new unmodifiable view of the provided set.

    **Parameters:**: `set` - the set for which an unmodifiable view is created.


**Method Detail**

### add

```
public boolean add(E o)
```

:   See Set.add(Object).

    :   **Specified by:**: `add` in interface `java.util.Collection<E>` **Specified by:**: `add` in interface `java.util.Set<E>`

---


### addAll

```
public boolean addAll(java.util.Collection<? extends E> c)
```

:   See Set.addAll(Collection).

    :   **Specified by:**: `addAll` in interface `java.util.Collection<E>` **Specified by:**: `addAll` in interface `java.util.Set<E>`

---


### clear

```
public void clear()
```

:   See Set.clear().

    :   **Specified by:**: `clear` in interface `java.util.Collection<E>` **Specified by:**: `clear` in interface `java.util.Set<E>`

---


### contains

```
public boolean contains(java.lang.Object o)
```

:   See Set.contains(Object).

    :   **Specified by:**: `contains` in interface `java.util.Collection<E>` **Specified by:**: `contains` in interface `java.util.Set<E>`

---


### containsAll

```
public boolean containsAll(java.util.Collection<?> c)
```

:   See Set.containsAll(Collection).

    :   **Specified by:**: `containsAll` in interface `java.util.Collection<E>` **Specified by:**: `containsAll` in interface `java.util.Set<E>`

---


### isEmpty

```
public boolean isEmpty()
```

:   See Set.isEmpty().

    :   **Specified by:**: `isEmpty` in interface `java.util.Collection<E>` **Specified by:**: `isEmpty` in interface `java.util.Set<E>`

---


### iterator

```
public java.util.Iterator<E> iterator()
```

:   See Set.iterator().

    :   **Specified by:**: `iterator` in interface `java.lang.Iterable<E>` **Specified by:**: `iterator` in interface `java.util.Collection<E>` **Specified by:**: `iterator` in interface `java.util.Set<E>`

---


### remove

```
public boolean remove(java.lang.Object o)
```

:   See Set.remove(Object).

    :   **Specified by:**: `remove` in interface `java.util.Collection<E>` **Specified by:**: `remove` in interface `java.util.Set<E>`

---


### removeAll

```
public boolean removeAll(java.util.Collection<?> c)
```

:   See Set.removeAll(Collection).

    :   **Specified by:**: `removeAll` in interface `java.util.Collection<E>` **Specified by:**: `removeAll` in interface `java.util.Set<E>`

---


### retainAll

```
public boolean retainAll(java.util.Collection<?> c)
```

:   See Set.retainAll(Collection).

    :   **Specified by:**: `retainAll` in interface `java.util.Collection<E>` **Specified by:**: `retainAll` in interface `java.util.Set<E>`

---


### size

```
public int size()
```

:   See Set.size().

    :   **Specified by:**: `size` in interface `java.util.Collection<E>` **Specified by:**: `size` in interface `java.util.Set<E>`

---


### toArray

```
public java.lang.Object[] toArray()
```

:   See Set.toArray().

    :   **Specified by:**: `toArray` in interface `java.util.Collection<E>` **Specified by:**: `toArray` in interface `java.util.Set<E>`

---


### toArray

```
public <T> T[] toArray(T[] a)
```

:   See Set.toArray(Object[]).

    :   **Specified by:**: `toArray` in interface `java.util.Collection<E>` **Specified by:**: `toArray` in interface `java.util.Set<E>`

---


### toString

```
public java.lang.String toString()
```

:   Returns `toString()` on the underlying set.

    :   **Overrides:**: `toString` in class `java.lang.Object`
    :   **Returns:**: `toString()` on the underlying set.

---


### equals

```
public boolean equals(java.lang.Object obj)
```

:   See Set.equals(Object).

    :   **Specified by:**: `equals` in interface `java.util.Collection<E>` **Specified by:**: `equals` in interface `java.util.Set<E>` **Overrides:**: `equals` in class `java.lang.Object`

---


### hashCode

```
public int hashCode()
```

:   See Set.hashCode().

    :   **Specified by:**: `hashCode` in interface `java.util.Collection<E>` **Specified by:**: `hashCode` in interface `java.util.Set<E>` **Overrides:**: `hashCode` in class `java.lang.Object`


---


|  |  |  |
| --- | --- | --- |
| **Overview** \| **Package** \| **Class** \| **Use** \| **Tree** \| **Deprecated** \| **Index** \| **Help** | |  |
| **PREV CLASS**   **NEXT CLASS** | **FRAMES**    **NO FRAMES**     **All Classes** |  |
| SUMMARY: NESTED \| FIELD \| CONSTR \| METHOD | DETAIL: FIELD \| CONSTR \| METHOD |  |


---
